# ‘Maybe they should regulate them quite strictly until they know the true dangers’: a focus group study exploring UK adolescents’ views on e‐cigarette regulation

**DOI:** 10.1111/add.13377

**Published:** 2016-04-18

**Authors:** Heide Weishaar, Filippo Trevisan, Shona Hilton

**Affiliations:** ^1^ MRC/CSO Social and Public Health Sciences Unit University of GlasgowUK; ^2^ School of Communication American UniversityWashington DCUSA

**Keywords:** Adolescents, age‐of‐sale, e‐cigarettes, focus groups, marketing, regulation, use in public places

## Abstract

**Background and aims:**

Regulation of electronic cigarettes has moved to the top of the addiction policy agenda, as demonstrated by the recent focus across the United Kingdom on introducing age‐of‐sale restrictions. However, the views of those affected by such regulation remain largely unexplored. This paper presents the first detailed qualitative exploration of adolescents’ perceptions of existing, and opinions about potential e‐cigarette regulation.

**Methods:**

Sixteen focus groups, including a total of 83 teenagers between the ages of 14 and 17 years, were conducted in deprived, mixed and affluent urban areas in Scotland and England between November 2014 and February 2015. Transcripts were imported into Nivivo 10, coded thematically and analysed.

**Results:**

Participants critically considered existing evidence and competing interests in regulatory debates and demonstrated sophisticated understanding of the advantages and disadvantages of regulation. They overwhelmingly supported strong e‐cigarette regulation and endorsed restrictions on sales to minors, marketing and e‐cigarette use in public places. Concern about potential health harms of e‐cigarette use and marketing increasing the acceptability of vaping and smoking led these adolescents to support regulation.

**Conclusions:**

In focus group discussions, a sample of UK adolescents exposed to particular communications about e‐cigarettes supported strict regulation of e‐cigarettes, including banning sales to minors and use in indoor public areas.

## Introduction

In the last few years the use of, and experimentation with, electronic cigarettes (e‐cigarettes) has risen sharply among adolescents 1, 2, 3. Although a causal relationship remains unclear, research suggests that adolescents who use e‐cigarettes might be more likely to take up smoking 4, 5, 6. These concerns have sparked regulatory actions (e.g. age‐of‐sales restrictions and regulation of advertising) by the European Union 7, English 8, Scottish 9 and Welsh 10 authorities.

Assessing whether policies are compatible with the views of those affected by them is important 11. Public opinion on, and acceptance of, existing and future public policies matter because levels of acceptability and support can critically affect policy effectiveness 12. Reviewing the opinion of target populations increases the chances of success of public health policy 13, and high levels of public support can enhance compliance 14. US research suggests that political decision makers are disposed to act in accordance with public opinion 15, possibly with a view to increasing their chances of re‐election 12. Therefore, public opinion can be a proximate and crucial factor of government action 12, 15, 16.

There is growing support in Europe, Australia, New Zealand, Canada and the United States for restricting smoking‐related behaviours, particularly when framed as interventions to protect children and adolescents 12. A better understanding of public opinion on e‐cigarette policies is crucial, as it can inform framing of, and communication about, existing and prospective policy initiatives, thereby increasing the acceptability of effective regulation 12, 13. Following good practice, the Scottish Government, the UK Department of Health, the Welsh Government and the European Commission held consultations aimed at gathering public opinion on e‐cigarette regulation. Strikingly, responses from organizations representing children and adolescents and individual representatives of this target group were largely absent from the consultations, indicating that the views of young people themselves have been insufficiently considered in this policy debate 13, 17. This paper explores adolescents’ perceptions of, and opinions about, e‐cigarette regulation. As the first in‐depth qualitative study to provide insights into adolescents' views of regulation of e‐cigarette sale, promotion and use, the paper sheds light on an unexplored area of e‐cigarette research, offering considerable potential to inform appropriate policy responses.

## Methods

We conducted 16 focus groups in Scotland (*n* = 11) and England (*n* = 5) between November 2014 and February 2015, i.e. prior to Scottish and English announcements of prospective e‐cigarette regulation. Focus groups included between four and seven participants (a total of 83 participants). Purposive sampling was used to recruit a diverse sample of adolescents in terms of gender, socio‐economic background, smoking status and e‐cigarette use. Participants for two groups were recruited directly by the researchers. Fourteen groups were recruited by local youth organizations through youth workers who had been briefed by the research team about the need to achieve gender balance, and include teenagers with a diverse range of experiences with regard to traditional and e‐cigarettes. Eleven of the 14 organizations that helped with participant recruitment worked specifically with young people from disadvantaged backgrounds in urban areas. This strategy resulted in the inclusion of a range of participants from more affluent and more deprived backgrounds and with experiences of smoking and vaping.

Focus group discussions were facilitated to allow the research team to explore how opinions about e‐cigarettes are developed and negotiated in peer‐led contexts. In order to avoid participants feeling pressured to agreeing with others, the following steps were taken. Participants were drawn from friendship groups to encourage open and frank discussions, and separate groups were held for 14–15 and 16–17‐year‐olds, respectively, to reduce the risk of older and younger participants influencing each other. Each participant was given a £20 shopping voucher as compensation for their time.

Prior to the start of the focus groups, participants completed a short anonymous questionnaire about their age, gender, smoking and e‐cigarette use status. For both traditional cigarettes and e‐cigarettes, the questionnaire asked participants to specify whether they had never tried, tried or used them in the past, or were using them at the time of the study. Patterns of use and experiences with traditional and e‐cigarettes were explored further in focus group discussions. Based on a review of the literature and pilot work, a topic guide was developed which covered five key areas, including: knowledge and understandings about e‐cigarettes; beliefs about the potential benefits, harms and associations of e‐cigarettes; experiences of e‐cigarette use; thoughts on the marketing of e‐cigarettes; and knowledge of, and opinions about, e‐cigarette regulation. Images of different types of e‐cigarettes (shisha pens, cig‐a‐likes and vape ‘tanks’) were used as conversation starters. In addition, promotional advertising material such as posters, still pictures from television and online advertisements were used to help stimulate group discussion about perceptions of e‐cigarettes. Group discussions were facilitated by an experienced researcher (F.T.), hosted by the youth organizations that helped with recruitment, and lasted between 40 and 70 minutes. Field‐notes, reflecting on the focus group and individual issues discussed, were written up for each group. All focus groups were audio recorded (with participants' permission) and transcribed verbatim. Each transcript was imported into NVivo 10, coded independently, cross‐checked and analysed by a team of six researchers. Codes, contradictory cases and group dynamics were discussed, making use of transcripts and field notes. Ethical approval for the study was obtained from the University of Glasgow's College of Social Sciences.

### The sample

Eighty‐three teenagers aged 14–17 years took part in the study. The study included 44 male (53%) and 39 female (47%) participants. Age distribution within the sample was skewed slightly towards 16–17‐year‐olds, with 17‐year‐olds making up the largest subgroup (*n* = 28). While the majority of participants did not currently smoke or use e‐cigarettes, the sample included 31 smokers and 47 adolescents who used e‐cigarettes or had used them in the past. E‐cigarette use and experimentation were distributed equally between males and females. Regular e‐cigarette users tended to be older. Smoking and e‐cigarette use among the sample are summarized in Table 1, and Table 2 describes the focus group composition and participants in more detail.

**Table 1:** E‐cigarette use according to cigarette smoking.

	E‐cigarette use											
	Never			Tried/Ex			Current				Total	
Cigarette smoker	n	(col %)	(row %)	n	(col %)	(row %)	n	(col %)	(row %)	n	(col %)	(row %)
Never	29	(81)	(78)	8	(22)	(22)	0	(0)	(0)	37	(45)	(100)
Tried/ex	6	(17)	(40)	9	(24)	(60)	0	(0)	(0)	15	(18)	(100)
Current	1	(3)	(3)	20	(54)	(65)	10	(100)	(32)	31	(37)	(100)
Total	36	(100)	(43)	37	(100)	(45)	10	(100)	(12)	83	(100)	(100)

**Table 2:** Focus group location, participants and their cigarette smoking and e‐cigarette use.

Location	Pseudonym	Age	Cigarette smoker	E‐cig use	Location	Pseudonym	Age	Cigarette smoker	E‐cig use
Glasgow Scotland	James	15	Never	Never	Edinburgh SE Scotland	Libby	16	Current	Tried/ex
	Gerry	14	Never	Never		Fergus	17	Current	Current
	Chrissie	14	Never	Never		Isaac	17	Never	Never
	Janice	15	Never	Never		Helen	17	Tried/ex	Never
Glasgow Scotland	Felicity	16	Never	Never		Matthew	17	Current	Tried/ex
	Donald	16	Never	Never	Kirkcaldy SE Scotland	Caiomhe	15	Never	Never
	Mitchell	16	Never	Never		Lauren	16	Never	Never
	Louis	16	Tried/ex	Never		Timothy	16	Current	Tried/ex
Glasgow Scotland	Thomas	16	Never	Never		Danyul	16	Current	Current
	Alice	17	Tried/ex	Tried/ex		Lesley	17	Tried/ex	Never
	Joshua	17	Never	Never		Jonathan	17	Never	Never
	Lachlan	16	Never	Never	Kirkcaldy SE Scotland	Fiona	16	Current	Current
	David	16	Tried/ex	Tried/ex		Scott	17	Current	Tried/ex
Glasgow Scotland	Robert	14	Never	Never		Murray	16	Current	Current
	Liam	14	Never	Tried/ex		Louisa	16	Current	Current
	Graham	14	Never	Never		Ella	16	Current	Current
	Adrian	15	Never	Never		Aisha	14	Current	Current
	John	14	Tried/ex	Never		Maggie	15	Current	Current
Glasgow Scotland	Maria	16	Never	Tried/ex	Greater ManchesterNW England	Rhyan	17	Tried/ex	Tried/ex
	Oliver	16	Never	Never		Charity	16	Never	Never
	Allan	17	Never	Never		Elizabeth	15	Never	Never
	Clare	17	Tried/ex	Tried/ex		Katie	17	Tried/ex	Never
	Jamie	17	Current	Never		Harriet	17	Never	Never
	Roslyn	17	Tried/ex	Tried/ex	Greater ManchesterNW England	Jane	16	Never	Never
Edinburgh Scotland	Iain	17	Current	Tried/ex		Darnell	14	Never	Tried/eEx
	Michael	17	Current	Tried/ex		George	16	Tried/ex	Tried/ex
	Stewart	17	Current	Current		Emma	17	Tried/ex	Tried/ex
	Richard	17	Current	Tried/ex		Paul	17	Never	Tried/ex
	Hannah	17	Current	Tried/ex	Merseyside NW England	Finlay	15	Never	Never
Edinburgh Scotland	Hayley	14	Current	Tried/ex		Sara	15	Never	Never
	Lucy	14	Current	Tried/ex		Steven	14	Never	Never
	Jennifer	14	Current	Tried/ex		Katherine	14	Never	Never
	Francis	14	Current	Tried/ex	Newcastle NE England	Fraser	14	Never	Tried/ex
	Judith	14	Current	Tried/ex		Susan	15	Never	Tried/ex
	Stuart	15	Never	Never		Karen	17	Tried/ex	Tried/ex
	Niall	14	Current	Tried/ex		Drew	16	Never	Tried/ex
Edinburgh SE Scotland	Lorna	16	Current	Tried/ex		Alex	17	Current	Tried/ex
	Sharon	16	Current	Tried/ex	Newcastle NE England	Rosie	17	Tried/ex	Never
	Ben	16	Current	Tried/ex		Carla	14	Tried/ex	Tried/ex
	Wendy	15	Current	Tried/ex		Mairi	15	Never	Tried/ex
	Christine	17	Current	Tried/ex		Gregor	17	Current	Current
						Henry	14	Never	Never

## Results

Overall, participants acknowledged that the regulation of e‐cigarettes required complex and ‘tricky’ decisions (17, male, non‐smoker, ex/tried e‐cigarette). One of the main factors that participants took into consideration when discussing e‐cigarette regulation was the lack of existing evidence about the potential short‐ and long‐term health risks both to consumers and bystanders:

I dinnae [do not] think they've been tested enough. […] Like, who's smoked vegetable oil through a tube, powered by a battery for ten year? Naebody's [Nobody has] done it yet. It's no' [not] been oot [out] that long, so nobody knows the long‐term effect (16, male, smoker, tried/ex e‐cigarette).

While aware of the lack of evidence and the difficulties in regulating a product whose harms were not fully known, many participants were supportive of strong regulation. Discourse about e‐cigarette regulation was dominated by comparisons between electronic and traditional cigarettes, with participants arguing that regulation of e‐cigarettes should be modelled on cigarette regulation. Another argument that was put forward to support strong regulation was that governments had a duty to protect citizens—particularly children—from harmful commodities, behaviours and addictive substances, including nicotine. Participants also demonstrated considerable awareness of the risk that regulation could prevent the use of e‐cigarettes as cessation devices and dual use, with regular or occasional smokers and dual users being particularly likely to draw attention to these concerns and the need to consider the situation of smokers, including smoking adolescents, when developing regulation. After balancing all arguments for and against regulation, the majority of participants, including smokers and e‐cigarette users, however, were generally supportive of precautionary measures to prevent potential harm connected with e‐cigarette use.

In 12 of 16 focus groups, participants spontaneously mentioned commercial interests of e‐cigarette manufacturers and retailers as important obstacles to adopting and implementing stringent regulation. Both e‐cigarette users and non‐users perceived commercial actors as unconcerned about the potential negative effects of their products. As a female non‐smoker and non‐e‐cigarette user put it, ‘as long as they're making money, they couldnae [could not] care about us' (16, female, never smoker, never e‐cigarette). Scepticism was also voiced with regard to manufacturers‘ alleged intentions to promote e‐cigarettes as smoking cessation aids. For example, a 17‐year‐old smoker who had tried e‐cigarettes claimed cynically that:

Not one of thae companies'll have a good intention tae [to] get you tae stop smoking. It's a good intention tae buy their product, and keep on buying (17, male, smoker, tried/ex e‐cigarette).

### Views on sale restrictions

Participants often seemed uncertain about the scope and nature of existing e‐cigarette regulation, in particular with regard to age‐of‐sale restrictions and e‐cigarette use in public places. Several participants thought that age‐of‐sale restrictions were in place, with many arguing that under‐18‐year‐olds were not allowed to purchase e‐cigarettes. Adolescents' reports which recalled that some shops prevented them from buying e‐cigarettes or required proof of identification and age verification seemed to reflect voluntary self‐regulation by some retailers. Despite occasional accounts of experiencing difficulties when trying to purchase e‐cigarettes, however, many participants reported that it was easy for them or their friends to obtain e‐cigarettes. E‐cigarette retailers’ economic interests were discussed as potential reasons for the inefficacy of self‐regulation, as outlined by the account of a 17‐year‐old male dual user:

The shop‐owner, he wouldnae [would not] care if he sold it tae somebody that's under 18 or that. Cos he needs tae sell them, ken [you know], like—he has tae make his money fae [from] somewhere (17, male, dual user).

Most participants were supportive of age‐of‐sale regulation. Opinions differed, however, on what age was considered appropriate for e‐cigarette purchases, with some participants favouring 18, whereas others suggested 16 or 14 as possible cut‐off ages. A popular argument for restricting sales to people aged 18 and over was that age restrictions on e‐cigarette sales should be aligned with sale restrictions on traditional cigarettes. Another prominent argument for some people for restricting sales to minors was that vaping was perceived as providing an avenue into smoking. Participants who were concerned about e‐cigarettes providing a gateway to smoking argued that while e‐cigarettes were suitable devices for smokers who were trying to quit, they were unsuitable for non‐smoking teenagers and should be regulated. While most participants, including most non‐smokers and non‐vapers, advocated for sale restrictions to be aligned with that of cigarettes, several smokers and dual users were in favour of lower cut‐off ages and argued that teenage smokers should be able to access less harmful alternatives to traditional cigarettes:

I'd say [e‐cigarettes should be available to] sixteen [year‐olds] ‘cause […] if you're smoking by then and there's a product that can actually help you and is cheaper than a normal just smoking, I don't see why no’ [not] (16, male, dual user).

Often, such arguments introduced conversations about the danger of undermining teenage quit attempts by restricting access to e‐cigarettes. Countering age‐of‐sale restrictions, a 17‐year‐old female dual user argued:

[In our] generation, younger people are smoking as well. Like, to try and stop, you're not able to. So it's a bit stupid that before, you were able tae buy cigarettes but you weren't allowed to buy something to help you stop (17, female, dual user).

Comprehensive restrictions on sales through the regulation of e‐cigarettes as medicines were discussed as alternative regulatory approaches to making e‐cigarettes available to smokers, including smoking teenagers. This option was discussed in three focus groups and voiced primarily by non‐smokers and non‐e‐cigarette users who argued that it would allow those who wanted to quit using e‐cigarettes to do so in a supervised and therefore ‘safer’ way.

I think it should be, like, prescribed, more of a medical thing, like, if you're trying to get adolescents to stop and they need something that'll kind of, like, wean them off it, rather than patches (15, female, never smoker, never e‐cigarette).

Discussions showed that participants perceived existing Medicines and Healthcare Products Regulatory Agency (MHRA) regulations as an official statement that medicinal e‐cigarettes were safe. The recent accreditation of one e‐cigarette as a medicinal product was discussed, with participants considering implications for users' perceptions of, and likelihood to use, the product:

There's one e‐cigarette that's been, like, verified by the government, is there not? […A news app] was saying something about the NHS [National Health Service] saying that they'd verified one being safe for use […] (16, male, smoker, ex/tried e‐cigarette).

If I knew that was safe, I would find that more attractive (yeah), like, if I knew for certain that there was nothing in it, you know, if it was government certified and everything I'd be, like: Right, buy it! (16, female, never smoker, never e‐cigarette).

### Views on e‐cigarette marketing restrictions

Participants were aware that ‘most shops now have [e‐cigarettes] right at the front o' the desk’ (16, female, dual user) which suggested rising pervasiveness and aggressiveness of e‐cigarette promotion. As illustrated by the following account of a 16‐year‐old male who had experimented with traditional and electronic cigarettes, participants demonstrated a clear awareness of promotional activities and sophisticated understanding of cigarette manufacturers’ strategic marketing:

[The target market for e‐cigarettes are] youth because then again, that opens up a market and if someone's becoming addicted when they're young [...] they'll have, like, a sort of loyal customer for many, many years, so that would be the reason that they're trying to put it in music videos and sort of current sort of pop songs and stuff like that (16, male, ex/tried smoker, ex/tried e‐cigarettes).

Despite such awareness, e‐cigarette marketing frequently seemed to have the anticipated success. A 17‐year‐old dual user, for example, reported that e‐cigarette promotion had prompted his use of e‐cigarettes.

It was a couple ae years ago, they were handing [disposable e‐cigarettes] out. Ken [you know], like, they were handing them oot for nothing. [...] But that's the first time I seen it. I was smoking it on the bus and everything. Ken [you know], I thought it was cool as fuck (17, male, dual user).

The majority of participants, and even smokers and e‐cigarette users, argued frequently that marketing restrictions of traditional cigarettes should extend to electronic cigarettes. Smokers, e‐cigarette users, non‐smokers and non‐vapers were also united around the view that children and young teenagers in particular should not be exposed to e‐cigarette marketing, with many participants discussing a 9 p.m. advertising watershed to prevent younger age groups from being lured into trying e‐cigarettes. The uncertainty about the health effects of e‐cigarettes was raised as a prominent argument for advertising bans. While participants acknowledged that a stronger evidence base was needed, they frequently favoured precautionary approaches and suggested that strict regulation should be pursued unless it was proven that e‐cigarettes were harmless. As the discussion below, between two male participants who neither smoked nor used e‐cigarettes shows, participants suggested that regulation could be revised once more evidence became available:

We don't know too much about e‐cigarettes. (Yeah) So maybe they should regulate them quite strictly until they know, like, the true dangers (17, male, never smoker, never e‐cigarette).

Yeah sure. (16, male, never smoker, never e‐cigarette).

And then if they do turn out healthy, they can start advertising (17, male, never smoker, never e‐cigarette).

Participants also argued that e‐cigarette marketing should be banned while opposition to regulation seemed relatively weak. For example, a 17‐year‐old male non‐smoker anticipated that, as the e‐cigarette market grew, manufacturers would defend their business interests more strongly by fighting marketing regulations:

If they banned, like, advertising in shops, like, now, it would be quite easy to sort of get rid of all the adverts and just, like, do away with them. But if they waited, like say, twenty years, and they became super‐popular, like, as big as cigarettes are now, it would be really hard fighting the, like, billion dollar companies and stuff to get all these bans enacted (17, male, never smoker, never e‐cigarette user).

When discussing potential regulation of e‐cigarette marketing, some smokers and e‐cigarette users highlighted that e‐cigarettes, if marketed, should be presented clearly as a product for smokers who wanted to quit or cut down and that marketing should include factual information and health warnings in order to increase awareness of the potential harms of the product. Several participants, including the following 15‐year‐old female dual user, were supportive of e‐cigarette marketing that endorsed non‐smoking and specifically targeted smokers to use e‐cigarettes as cessation aid:

If it does help people cut down and stuff like that [then advertising should be allowed] […] It should maybe make [e‐cigarette advertising] more like anti‐smoking and this is how you could stop smoking rather than making it out to seem like it's like a really good thing. (15 female, dual user).

While participants perceived the long‐standing ban on advertising of traditional cigarettes as a strong official statement about the health hazards of cigarettes, allowing marketing of e‐cigarettes was understood as an implicit government endorsement of the products. A 16‐year‐old male, who had experimented with both traditional and e‐cigarettes, hypothesized that viewers of e‐cigarette advertisements might think that e‐cigarettes ‘are fine, 'cause they're advertising [them], so the government thinks they're alright’ (16, male, ex/tried smoker, ex/tried e‐cigarette).

### Views on the use of e‐cigarettes in public and private places

The use of e‐cigarettes in public places and restrictions thereof were a broadly debated issue in all focus groups. The inconsistency of existing restrictions in different venues seemed to contribute to confusion about the places where vaping was allowed. While participants reported uniformly that e‐cigarette use was forbidden on aeroplanes, they were less clear about regulation on other public transport and in hospitality venues. Participants also reported that inconsistent and voluntary regulation led to lack of enforcement and exploitation of loopholes:

In some places, they've put rules against it but it's not the law (yeah), so people can still sort of sneakily bring them out, have a couple of puffs on them, no need to go outdoors. But it's a sort of, it's like, it's a grey area right now (unidentified male).

Similarly, participants reported that vaping was not allowed in their schools, but explained that students ‘don't really abide by that rule' (17, male, never smoker, ex/tried e‐cigarette). Mirroring multiple accounts of vaping in schools, a 16‐year‐old female never smoker and never e‐cigarette user reported that ‘because there's like no cigarette smell and stuff, they're more easier to hide than actual cigarettes’ (16, female, never smoker, never e‐cigarette).

Five non‐smokers and non‐e‐cigarette users and one dual user were critical of vaping bans in public places, citing the lack of evidence of the harms of second‐hand vapour, particularly when compared to second‐hand smoke and perceiving public vaping to not be an issue to tackle. Most participants, including the majority of smokers and e‐cigarette users, however, were supportive of banning e‐cigarette use in public places and felt that vaping should be as equally regulated as smoking. Key rationales for this were the adverse effects and potential harms caused by second‐hand vapour on bystanders. Most concerns centred around the potential negative impact on children who were perceived as particularly vulnerable to health harms and easily influenced by seeing adults using cigarette‐like devices. Participants were concerned that vaping in public places might contribute to the (re‐)normalization of both e‐cigarette and traditional cigarette use. A 14‐year‐old female never smoker and never e‐cigarette user illustrated this by saying:

Younger people are going to see [people using e‐cigarettes] and, like, wonder what it is and maybe try it, and it can lead on from there (14, female, never smoker, never e‐cigarette).

Consequentially, the use of e‐cigarettes in public places was recognized as a type of ‘free advertising to adolescents’ (16, female, never smoker, never e‐cigarette).

Reflecting contemporary debates on smoking in private places, some participants considered the possibility of restricting e‐cigarette use in private places where children were present. Arguments that had been promoted publicly to support restrictions on smoking in cars with children present were reiterated, including the potential harms of e‐cigarette use to children and the inability of children to protect themselves in the confined space of a car. The harms of vaping in front of children in the private home was also highlighted, with participants apparently aware of ongoing regional awareness campaigns about effective protection from second‐hand smoke in the home and the need to smoke at a distance of at least ‘seven steps outside of your house’ (17, male, ex/tried smoker, ex/tried e‐cigarette).

## Discussion

E‐cigarettes have become increasingly popular and visible in public life. In response to potential health harms, policymakers are increasingly contemplating regulation. While public consultations capture information on adults’ opinions about e‐cigarette regulation, the views of adolescents, a key target population of legislation, have so far remained unexplored. To the best of our knowledge, this paper is the first to present detailed insights of adolescents' awareness of, and opinions about, e‐cigarette regulation.

Our analysis shows that the adolescent participants in this study had a sophisticated understanding of the potential advantages (e.g. increased attempts to quit smoking) and disadvantages (e.g. re‐normalization of smoking‐like behaviour, increased attractiveness of a potentially harmful product, socialization of adolescents into nicotine consumption) of e‐cigarette use and marketing, considered the existing evidence base critically and showed ample awareness of the difficulties of making regulatory decisions in view of inconsistent and inconclusive evidence of long‐term harms. They demonstrated a good understanding of the competing interests that influence debates on e‐cigarette regulation and the potentially detrimental effects of commercial interests on the development and implementation of effective policy. Overall, UK adolescents seemed to be largely supportive of governmental regulation to restrict e‐cigarette sale, promotion and use, and even of policies which restrict their own access to, and use of, e‐cigarettes. They particularly endorsed age‐of‐sale restrictions, restraints on marketing targeted at adolescents and non‐smokers and the use of e‐cigarettes in public places where children and young teenagers are present, thereby going beyond calls of some public health organizations 18, 19.

Our findings are in line with previous research which highlights strong public support for alcohol and tobacco regulation 20, and show that adolescents were unlikely to perceive public health legislation as an intrusion into their individual freedom and autonomy. In fact, our results indicate that adolescents see the protection of citizens from the harms caused by e‐cigarettes as a governmental duty. Support for regulation seems to derive from adolescents' concerns about the potential harms of e‐cigarettes to both e‐cigarette users and bystanders, with a notable number of concerns focusing specifically on children's health. The fact that adolescents often focused on discussing regulation as a sensible tool to restrict e‐cigarette access of, promotion to, and use among, younger teenagers and children suggests that 14–17‐year‐olds did not always perceive themselves as the primary target group of e‐cigarette regulation and might be indicative of some degree of a ‘third person effect’ 21. The analysis, however, suggests overall that UK adolescents, including e‐cigarette users, did not reject e‐cigarette regulation which applies to themselves and restricts their own access to, and use of, these products. The findings show that adolescents who smoked or engaged in dual use were concerned about the particular needs of teenage smokers who intend to quit and raised considerations about the potential benefits of e‐cigarettes for this population group. If future research was to reveal that e‐cigarette use causes minimal risks and increases quit rates significantly, policy developments might have to consider carefully how to facilitate e‐cigarette access for adolescent smokers while not undermining or disregarding existing adolescent support for stringent regulation of these products.

UK adolescents often seemed unclear about existing e‐cigarette regulation, particularly with regard to age‐of‐sale restrictions and the use of e‐cigarettes in public places. The data provide strong evidence that inconsistency of restrictions contributed to adolescents' lack of understanding of, and uncertainty about, statutory regulation of e‐cigarettes. By highlighting that adolescents perceived governmental regulation as an endorsement, and lack thereof as disapproval of the products, the analysis indicates that UK adolescents are in need of reliable information about e‐cigarette regulation and that strong, consistent rules have the potential to communicate powerful prevention messages.

There are several limitations to our study. First, and consistent with the qualitative design, the sample does not aim to be representative of UK youth. Secondly, the study's geographical remit has to be considered when interpreting the findings. The United Kingdom is an international leader in tobacco control policy 22, and participants' views were probably influenced by the UK's legal and socio‐cultural context, including low smoking prevalence and high acceptability of tobacco control measures. Whether adolescents' support of e‐cigarette regulation is equally high in legislatures with weaker tobacco control laws remains to be explored. Thirdly, in accordance with previous studies which have shown that the views on regulation of consumers of addictive substances differ by respondents' characteristics 12, 13, 23, we conducted a basic analysis of differences in opinions with regard to smoking status and e‐cigarette use. More detailed analyses accounting for correlations between other socio‐economic variables and adolescent opinions on e‐cigarette regulation remain outstanding.

Despite such limitations, this study considerably increases understanding of UK adolescents' views on e‐cigarette regulation. As outlined above, arguably the most interesting finding is that adolescent smokers, e‐cigarette users, non‐smokers and non‐e‐cigarette users were united in their support of stringent e‐cigarette regulation. Fairchild & Bayer distinguish between harm reduction and precautionary approaches to e‐cigarette regulation 24, and a similar division has occurred in recent ‘fierce ideological debate[s]' 25 on e‐cigarettes, with some advocates arguing against ‘excessive restrictions' 26 and others advocating for strong regulatory frameworks 27. Our analysis suggests that UK adolescents, while aware of the potential of e‐cigarettes to assist smokers in quitting, overwhelmingly supported precautionary approaches and favoured regulation that guards against as yet underexplored impacts of e‐cigarette use on non‐smokers, especially children and young teenagers. Our findings thus echo the 2014 Scottish Youth Commission on Smoking Prevention report to the Scottish Government which highlights that Scottish youth are in favour of aligning e‐cigarette regulation with tobacco product regulation 28. The findings also support increasing calls by scholars 29, 30 and policymakers 31, 32 which highlight the urgent need and public support for strong regulation to protect children and adolescents effectively from the potential detrimental impact of increasing e‐cigarette marketing and consumption and the risks of socialization into new forms of nicotine use. The latter is a particular concern, in light of the harmful effects of nicotine on the adolescent brain and the relative lack of attention to this issue in current public and political debates 33. A challenge for policymakers is thus to develop regulation which takes into account adolescent support for strong regulation, prevents initiation and growth of smoking‐like behaviour and nicotine use among non‐smoking adolescents and helps smoking adolescents to quit effectively.

Previous research suggests that the impact of public opinion on policy is substantial 11, and that public attitudes and norms about regulation influence policymakers’ disposition to adopt tobacco control policies 34. Our analysis suggests that future UK age‐of‐sale restrictions on e‐cigarettes will be welcomed by adolescents. Perhaps more importantly, our study indicates that even additional regulation of e‐cigarette marketing and public use is likely to meet with strong support from this generation.

## Declaration of interest

None.
